# Effects of dynamic zero COVID-19 policy on anxiety status and lifestyle changes of pregnant women in rural South China: a survey-based analysis by propensity score matching method

**DOI:** 10.3389/fpubh.2023.1182619

**Published:** 2023-06-22

**Authors:** Ye Ding, Xi Shi, Genyuan Li, Qingfen Liang, Ziqi Yang, Yanxia Peng, Huiqin Deng, Zhixu Wang

**Affiliations:** ^1^Department of Maternal, Child and Adolescent Health, School of Public Health, Nanjing Medical University, Nanjing, China; ^2^Lingshan Maternal and Child Health Hospital, Qinzhou, China; ^3^Tianyang Maternal and Child Health Hospital, Baise, China; ^4^Zijin Maternal and Child Health Hospital, Heyuan, China; ^5^Longchuan Maternal and Child Health Hospital, Heyuan, China

**Keywords:** dynamic zero COVID-19 policy, rural areas, pregnant women, anxiety, lifestyles

## Abstract

**Introduction:**

The coronavirus disease 2019 (COVID-19) pandemic triggered a global public health crisis and has brought an unprecedented impact on pregnant women. The problems faced by pregnant women in the rural areas of China during the epidemic are different from those in urban areas. Although the epidemic situation in China has gradually improved, studying the impact of the previous dynamic zero COVID-19 policy on the anxiety status and lifestyle of pregnant women in rural areas of China, is still necessary.

**Methods:**

A cross-sectional survey of pregnant women in rural South China was conducted from September 2021 to June 2022.Using questionnaires, sociodemographic characteristics, anxiety status, physical activity, sleep quality, and dietary status of the population were collected. Using the propensity score matching method, the effect of the dynamic zero COVID-19 strategy on the anxiety status and lifestyle of pregnant women was analyzed.

**Results:**

Among the pregnant women in the policy group (*n* = 136) and the control group (*n* = 680), 25.7 and 22.4% had anxiety disorders, 83.1 and 84.7% had low or medium levels of physical activity, and 28.7 and 29.1% had sleep disorders, respectively. However, no significant difference (*p* > 0.05) was observed between the two groups. Compared with control group, the intake of fruit in the policy group increased significantly (*p* = 0.019), whereas that of aquatic products and eggs decreased significantly (*p* = 0.027). Both groups exhibited an unreasonable dietary structure and poor compliance with the Chinese dietary guidelines for pregnant women (*p* > 0.05). The proportion of pregnant women in the policy group, whose intake of stable food (*p* = 0.002), soybean, and nuts (*p* = 0.004) was less than the recommended amount, was significantly higher than that in the control group.

**Discussion:**

The dynamic zero COVID-19 strategy had little impact on the anxiety status, physical activity, and sleep disorders of pregnant women in the rural areas of South China. However, it affected their intake of certain food groups. Improving corresponding food supply and organized nutritional support should be addressed as a strategic approach to improve the health of pregnant women in rural South China during the pandemic.

## 1. Introduction

The coronavirus disease 2019 (COVID-19) pandemic triggered a global public health crisis and has brought an unprecedented impact on the health, economy, and society on the entire human population (1). According to the statistics published on the WHO website, as of December 2022, the number of patients with COVID-19 worldwide reached 730 million, with a mortality rate of over 0.92% (2). With the global spread of the epidemic, its harm to people was no longer limited to the direct impact of the virus itself, but also included the health impacts of psychological changes, medical delays, reduced exercise, and other changes caused by the epidemic. Pregnancy is a special stage in a woman’s life. Studies showed that, compared with non-pregnant women, pregnant women infected with COVID-19 had a higher risk of entering the ICU and receiving mechanical ventilation (3, 4) and were associated with an increased risk of maternal and neonatal complications, such as preeclampsia, miscarriage, preterm birth, intrauterine growth restriction, and fetal distress (5, 6). So far, the direct threat of COVID-19 to pregnant women’s health has been widely concerned, but its indirect threat to pregnant women still needs further evaluation. Due to changes in hormones, concerns about fetal growth, and fear of pain caused by childbirth, the emotional sensitivity of pregnant women (especially primiparous women) fluctuates greatly. Studies have found that problems such as the risk of COVID-19 infection, inconvenience of medical services, and irritability induced by access being limited to the home aggravated the anxiety of pregnant women and even increased mental illness prevalence (7, 8). This epidemic also significantly affected their physical activity, sleep, and diet, which further affected maternal and infant health (9). For example, studies in Japan and Poland have found that the pandemic may have affected the physical activity level or even accelerated physical inactivity (9, 10). Because of the decrease in activity time, sleep rhythm disorders occurred, and the risk of sleep disorders increased (11). Some studies comparing the dietary consumption of pregnant women before and during the COVID-19 pandemic have shown that the intake of vegetables, fruits, dairy products, fish, and legumes decreased (1, 9).

The rural population of China is huge (approximately 500 million) (12). Compared with cities with large population mobility, rural areas have a relatively fixed population, which is advantageous for epidemic prevention and control. In the early stage, average COVID-19 infection rates were lower in rural areas than in urban areas. However, with the spread of the epidemic, COVID-19 had hit rural residents considerably harder than urban residents (13). In rural areas, information is not available in time, medical resources are scarce, and medical conditions are worse compared with those in cities (14, 15). Houses in rural areas are scattered and the roads extend in all directions. Therefore, implementing blockade and quarantine measures in rural areas is not as convenient as in urban areas. Because of the low health literacy level and weak awareness of prevention, rural people wear masks considerably less frequently than urban residents (16). In addition, the shops in rural areas are relatively limited. Many shops are closed during the epidemic, and people have no suitable goods to choose from (9). Based on these particularities, studying the changes in the psychology, physical activity, sleep, and diet of pregnant women in rural China during the epidemic is necessary. However, the relevant research is relatively limited.

With an increase in vaccine coverage and the availability of specific drugs, the epidemic prevention and control measures in China have been adjusted. From August 2021, the dynamic zero COVID-19 policy of full-chain precise prevention and control has been adopted. When COVID-19 cases occur, effective and comprehensive prevention and control measures will be taken to quickly cut off the transmission chain of the epidemic, so that each epidemic can end in time, the number of infected people will be “zero “, and the maximum effect will be achieved at the lowest cost (17, 18). The specific measures include three aspects: first, timely and active detection of the infection source, mainly by mon-itoring the early warning of fever clinics and by using some rapid detection and screening methods, such as antigen and nucleic acid detection, after the collection of nasal and throat swabs. Second, when cases are found, public health and social intervention measures need to be taken quickly, including control of the outbreak point, management of close contacts, epidemiological investigation, and reduction of crowd gathering. Third, patients must be effectively treated, mainly by combining traditional Chinese and western medicine therapies, thus aiming to immediately stop the progress of the epidemic, prevent the disease from worsening, and reduce the occurrence of severe cases and death (19).

Although Chinese people have gradually returned to normal life, whether the dynamic zero COVID-19 policy has affected the psychological, physical activity, sleep, and diet of pregnant women in the rural areas of China remains to be explored. Because the pandemic is not over yet, people may still follow certain behaviors to deal with COVID-19. We here conducted a cross-sectional survey on some pregnant women in rural South China and analyzed the effect of the dynamic zero COVID-19 strategy on the anxiety status and lifestyle of pregnant women based on the propensity score matching (PSM) method.

## 2. Methods

### 2.1. Study design and participants

This cross-sectional survey was conducted from September 2021 to June 2022 (the stage of the dynamic zero COVID-19 policy). The South China region is one of the seven major geographical regions in China, including Guangdong Province, Guangxi Zhuang Autonomous Region, Hainan Province, Hong Kong Special Administrative Region, and Macau Special Administrative Region. A multi-stage sampling method was employed to enroll the study participants. In the first stage, Guangdong Province and Guangxi Zhuang Autonomous Region were selected as representatives according to the geographical location and convenience of implementation. In the second stage, each province (or autonomous region) was divided into urban and rural areas. Finally, two maternal and child health care institutions were randomly selected from the rural areas of each province (or autonomous region). Pregnant women aged 18–49 who had resided locally for more than 12 months were recruited at these hospitals. Women with speech communication difficulties or mental disorders were excluded. All women provided their signed informed consent. The study was approved by the Ethics Committee of Tongji Medical College, Huazhong University of Science and Technology (No. 2021-S092).

### 2.2. Data collection and measures

In order to study the impact of the previous dynamic zero COVID-19 policy on the anxiety status and lifestyle of rural pregnant women, they were divided into policy group and control group. According to the government risk area demarcation, pregnant women with overlapping home addresses and risk areas were in the policy group and the remaining were in the control group. Pregnant women in the policy group obeyed the rules of the dynamic zero COVID-19 policy. None of the two groups were infected with COVID-19. The data on demographic characteristics, anxiety, physical activity, and sleep quality were collected through a face-to-face questionnaire survey, and the dietary status of pregnant women were evaluated using a semi-quantitative food frequency questionnaire (FFQ). Of the original sample, 1,386 pregnant women were included. In order to eliminate the influence of confounding factors on the study results, we employed the nearest neighbor matching of the PSM method (20) based on the caliper value <0.02. The covariate factors used for matching included the pregnancy stage, age, ethnicity, education, income, and parity. Owing to the difficulties in the survey of pregnant women in the risk area and to improve the statistical power, a 1: 5 matching method was adopted. Finally, 136 women (16.7%) in the policy group and 680 women (83.3%) in the control group were enrolled. The covariates were balanced after matching, and the differences in the sociodemographic characteristics between the two groups before and after matching are presented in Table 1.

**Table 1:** Sociodemographic characteristics of pregnant women before and after propensity score matching.

Characteristics	Before matching			After matching		
	Policy (*n* = 136)	Control (*n* = 1,250)	*p*	Policy (*n* = 136)	Control (*n* = 680)	*p*
Pregnancy stage			0.873			0.750
First trimester, *n* (%)	42 (30.9)	407 (32.6)		42 (30.9)	227 (33.4)	
Second trimester, *n* (%)	51 (37.5)	442 (35.4)		51 (37.5)	233 (34.3)	
Third trimester, *n* (%)	43 (31.6)	401 (32.0)		43 (31.6)	220 (32.3)	
Age (years), mean ± SD	29.7 ± 5.9	28.8 ± 5.1	0.088	29.7 ± 5.9	29.4 ± 5.2	0.631
Height (cm), mean ± SD	156.3 ± 4.6	156.5 ± 5.0	0.696	156.3 ± 4.6	156.6 ± 4.9	0.572
Pre-pregnancy BMI (kg/m^2^), mean ± SD	20.9 ± 2.9	21.0 ± 3.4	0.511	20.9 ± 2.9	21.0 ± 3.3	0.685
Current BMI (kg/m^2^), mean ± SD	23.5 ± 3.3	23.4 ± 3.7	0.576	24.8 ± 3.6	23.0 ± 3.5	0.140
Ethnicity			<0.001			0.628
Han, *n* (%)	82 (60.3)	933 (74.6)		82 (60.3)	425 (62.5)	
Minority, *n* (%)	54 (39.7)	317 (25.4)		54 (39.7)	255 (37.5)	
Educational level			0.068			0.898
Junior high school and below, n (%)	54 (39.7)	599 (47.9)		54 (39.7)	274 (40.3)	
Junior high school above, n (%)	82 (60.3)	651 (52.1)		82 (60.3)	406 (59.7)	
Occupation			0.911			0.798
Housewife, *n* (%)	83 (61.0)	769 (61.5)		83 (61.0)	407 (59.9)	
Working, *n* (%)	53 (39.0)	481 (38.5)		53 (39.0)	273 (40.1)	
Monthly income (RMB)			0.129			0.282
<3,000, *n* (%)	64 (47.1)	504 (40.3)		64 (47.1)	286 (42.1)	
≥3,000, *n* (%)	72 (52.9)	746 (59.7)		72 (52.9)	394 (57.9)	
Parity			0.622			0.612
Primiparous, *n* (%)	55 (40.4)	533 (42.6)		55 (40.4)	291 (42.8)	
Multiparous, *n* (%)	81 (59.6)	717 (57.4)		81 (59.6)	389 (57.2)	

SD, standard deviation; BMI, body mass index.

#### 2.2.1. Questionnaires used to collect anxiety status, physical activity, and sleep quality

The Self-rating Anxiety Scale (SAS) used in this study was developed by Zung in 1971 (21) to assess the anxiety status of pregnant women in the past week. The correlation coefficient between this scale and the Hamilton Self-Rating Anxiety Scale was 0.37 (22). SAS consisted of 20 items. According to the frequency of symptoms, each item was categorized into 4 grades. Among them, 15 were positive scores and 5 were negative scores. The sum of the scores of 20 items was the total rough score, and the latter was multiplied by 1.25, which was the total standard score. In this study, a total standard score < 50 was considered to indicate a normal condition, and a total standard score ≥ 50 was considered to indicate anxiety disorder, among which the score of 50–62 was considered to indicate mild anxiety disorder, while a score > 62 was considered to indicate a severe anxiety disorder (23).

The short version of the International Physical Activity Questionnaire (IPAQ) was applied to measure the physical activity level of pregnant women in the past week. The questionnaire consisted of 7 items related to the frequency and duration of weekly walking, moderate-intensity, and vigorous-intensity physical activities. The reliability and validity of the Chinese version of IPAQ were tested. The reliability coefficient was found to be 0.66–0.89, and the validity coefficient was 0.60–0.78 (24). In this study, ac-cording to the energy requirements defined in metabolic equivalent (MET), these ac-tivities were weighted to generate a MET-minute score, which was then computed by multiplying the MET score with the minutes performed (walking = 3.3 METs, moderate activity = 4.0 METs, and vigorous activity = 8.0 METs). The total physical activity level (MET-min/week) of pregnant women was calculated and then divided into low, medium, and high intensities (25).

The Pittsburgh Sleep Quality Index (PSQI) proposed by Buysse (26) in 1989 was referred to evaluate the sleep quality of pregnant women in the past month. The reliability and validity of the Chinese version of PSQI were tested in Chinese adults, and the results indicated that the split-half reliability coefficient was 0.82 and the overall Cronbach’s α-coefficient was 0.85 (27). In this study, PSQI consisted of 18 self-reported items, which were divided into 7 subcategories, as given below: subjective sleep quality, sleep latency, sleep duration, habitual sleep efficiency, sleep disturbances, use of sleeping medication, and daytime dysfunction. Each subcategory was scored 0–3. The total PSQI score ranged from 0 to 21, with higher scores indicating poorer sleep quality. PSQI ≤4 (good sleep), 4 < PSQI <8 (general sleep), and PSQI ≥8 (sleep disorders) served as the criteria for judging sleep quality (28).

#### 2.2.2. Food intake questionnaire and dietary data analysis

A semi-quantitative FFQ was used to investigate the dietary status of pregnant women in the past month. This FFQ includes the following 3 portions: the food list, the frequency of eating a certain food, and the amount of each consumption. There were 61 items on the food list, which were then divided into the following 13 categories: staple food (cereals and their products, potatoes, and beans other than soybeans); vegetables; fruits; livestock meat and poultry; aquatic products (fish, shrimp, and shellfish); eggs; milk and its products; soybean and its products; nuts; cooking oil; processed food; flavorings; beverages. It was specially designed for pregnant women and was validated against three 24-h dietary recalls. For foods, the intraclass correlation coefficients of two administrations of FFQ ranged from 0.23 (nuts) to 0.49 (fruits), and the energy-adjusted and de-attenuated correlation coefficients between the 2 methods ranged from 0.35 (beans) to 0.56 (fruits) (29). To improve the accuracy of food-weight estimation, tableware and the food atlas developed by our research team were integrated into the dietary intake recall (30).

The raw data on the amount of food was input into EpiData software for verification. The daily food intake of each group was then calculated. The food intakes of the 9 main food groups (i.e., staple food; vegetables; fruits; livestock meat and poultry; aquatic products; eggs; milk and its products; soybean and its products; nuts) were compared with the recommended intakes of the Chinese balanced dietary pagoda for pregnant women (31). The number of pregnant women within and out of the recommended intake ranges was recorded. The Chinese Dietary Guidelines Compliance Index for Pregnant Women (CDGCI-PW) was further used to assess the overall dietary status of pregnant women. This index was developed by our research group (32) and included 13 components, with a total score of 100 points. The CDGCI-PW score reflected the compliance of the pregnant women with the Chinese dietary guidelines for pregnant women. The higher the CDGCI-PW score, the better the dietary quality.

### 2.3. Statistical analysis

SPSS V.24 was used for statistical analysis. Normally distributed continuous variables were expressed as the mean ± standard deviation (SD) and compared via Student’s *t*-test. Non-normally distributed continuous variables were ex-pressed as median (interquartile range) and analyzed by the Mann–Whitney *U*-test. Categorical variables were expressed as frequency (*n*) and percentage (%) and analyzed using the Chi-square test or Mann–Whitney *U*-test. *p* < 0.05 was considered to indicate a statistically significant difference.

## 3. Results

### 3.1. Sociodemographic characteristics of pregnant women

As shown in Table 1, before PSM, a significant difference in ethnic distribution was observed between the policy and control groups. After PSM, no statistically significant difference in baseline characteristics was observed between the two groups, indicating that the propensity scores were well-matched. In total, 816 pregnant women (mean age: 29.5 ± 5.5 years, mean height: 156.4 ± 5.1 cm, mean current body mass index (BMI): 23.3 ± 3.6 kg/m^2^) were included in the analysis. Among them, 34.8 and 32.2% of pregnant women were in the second and third trimesters, respectively. Of the pregnant women, 62.1% were Han nationality, 59.8% had an education level above junior high school, 60.0% were housewives, 46.3% had a *per capita* income of 3,000 yuan/month or more, and 57.6% were multiparous.

### 3.2. Anxiety status, physical activity, and sleep quality of pregnant women

The SAS scores of the policy and control groups were 46.3 ± 6.0 and 45.6 ± 5.4, respectively. In the two groups, 34 (25.0%) and 150 (22.1%) pregnant women had mild anxiety disorder, while 1 (0.7%) and 2 (0.3%) pregnant women had severe anxiety disorder (Table 2). Further statistical analysis revealed no significant difference in anxiety status between the two groups (*p* = 0.771 and *p* = 0.380).

**Table 2:** Comparison of the anxiety status, physical activity, and sleep quality of pregnant women between the two study groups.

Lifestyles	Item	Policy (*n* = 136)	Control (*n* = 680)	*p*
Anxiety status	SAS score, mean ± SD	46.3 ± 6.0	45.6 ± 5.4	0.771
	Anxiety levels, *n* (%)			0.380
	Normal	101 (74.3)	528 (77.6)	
	Mild	34 (25.0)	150 (22.1)	
	Severe	1 (0.7)	2 (0.3)	
Physical activity	Physical activity score, MET-min/week, mean ± SD	1942.8 ± 1521.4	1952.3 ± 1563.2	0.213
	Physical activity levels, n(%)			0.371
	Low	40 (29.4)	156 (22.9)	
	Medium	73 (53.7)	420 (61.8)	
	High	23 (16.9)	104 (15.3)	
Sleep quality	PSQI score, mean ± SD	6.0 ± 3.1	6.1 ± 3.4	0.948
	Sleep Quality, n (%)			0.897
	Good	48 (35.3)	249 (36.6)	
	General	49 (36.0)	233 (34.3)	
	Sleep disorders	39 (28.7)	198 (29.1)	

SAS, Self-rating Anxiety Scale; SD, standard deviation; MET: Metabolic Equivalent Task; PSQI, Pittsburgh Sleep Quality Index.

The physical activity scores of the policy and control groups were 1942.8 ± 1521.4 MET-min/week and 1952.3 ± 1563.2 MET-min/week, respectively, and there was no statistically significant difference between the two groups (*p* = 0.213). After being divided into different intensities based on scores, there were 40 (29.4%) and 156 (22.9%) pregnant women with low levels of physical activity, and 73 (53.7%) and 420 (61.8%) pregnant women with medium levels of physical activity in the policy group and control groups, respectively, with no statistically significant difference (*p* = 0.371).

The PSQI scores of the policy and control groups were 6.0 ± 3.1 and 6.1 ± 3.4, respectively, and 39 (28.7%) and 198 (29.1%) pregnant women in the two groups had sleep disorders, respectively. Statistical analysis showed that there was no significant difference in the PSQI score and prevalence of sleep disorders between the two groups (*p* = 0.948 and *p* = 0.897).

### 3.3. Food intake of pregnant women

As shown in Table 3, compared with the control group, fruit intake in the policy group was significantly increased (300.0 > 260.0 g/day, *p* = 0.019), whereas the intake of aquatic products (30.6 < 41.6 g/day, *p* = 0.027) and eggs (28.6 < 34.3 g/day, *p* = 0.034) decreased significantly. The intake of other food groups, namely staple foods, vegetables, livestock meat and poultry, milk and its products, soybean and its products, and nuts, was not statistically significant between the two groups (*p* > 0.05).

**Table 3:** Comparison of food intake (g/day) of the pregnant women between the two study groups.

Food groups	Policy (*n* = 136)	Control (*n* = 680)	*p*
Staple food	258.3 (223.6, 280.5)	268.9.0 (216.4, 336.2)	0.152
Vegetables	408.1 (284.8, 527.1)	373.3 (250.0, 500.0)	0.057
Fruits	300.0 (200.0, 450.0)	260.0 (180.0, 385.7)	0.019
Livestock and poultry	88.9 (50.7, 137.0)	85.8 (57.1, 154.3)	0.345
Aquatic products	30.6 (7.1, 71.4)	41.6 (16.0, 71.4)	0.027
Eggs	28.6 (14.3, 50.0)	34.3 (16.7, 50.0)	0.034
Milk and its products	114.0 (17.0, 250.0)	142.9 (8.6, 250.0)	0.379
Soybean and its products	10.0 (2.3, 16.1)	10.0 (4.3, 17.6)	0.097
Nuts	2.6 (0.0, 10.0)	4.0 (0.0, 17.1)	0.106
CDGCI-PW	54.9 ± 12.5	55.9 ± 13.2	0.392

CDGCI-PW, Chinese Dietary Guidelines Compliance Index for Pregnant Women. Data are presented as median (P25; P75).

In comparison with the corresponding recommended intake in the Chinese balanced dietary pagoda for pregnant women, the dietary structure of both groups was found to be unreasonable. As shown in Table 4, the main problems were concentrated in the large proportion of pregnant women with insufficient intake of milk and its products, soybean, and nuts. Moreover, statistical analysis revealed that the proportion of pregnant women in the policy group who consumed less than the recommended amounts of stable food (*p* = 0.002), soybean, and nuts (*p* = 0.004) was statistically higher than that in the control group. The intakes of other food groups, namely vegetables, fruits, livestock meat and poultry, aquatic products and eggs, and milk and its products, were not statistically significant between the two groups (*p* > 0.05).

**Table 4:** Comparison of the recommended values and the actual food intake by pregnant women from the policy and control groups, *n* (%).

Food groups	Policy (*n* = 136)			Control (*n* = 680)			*p*	Rec, g/d
	Below rec^a^	Rec^b^	Above rec^c^	Below rec^a^	Rec^b^	Above rec^c^		
Staple food	60 (44.1)	57 (41.9)	19 (14.0)	258 (37.9)	198 (29.1)	224 (33.0)	0.002	Frist trimester 250 ~ 300 Second trimester 275 ~ 325 Third trimester 300 ~ 350
Vegetables	38 (27.9)	57 (41.9)	41 (30.2)	234 (34.4)	290 (42.6)	156 (23.0)	0.055	Frist trimester 300 ~ 500 Second trimester 300 ~ 500 Third trimester 300 ~ 500
Fruits	58 (42.6)	52 (38.2)	26 (19.2)	300 (44.1)	257 (37.8)	123 (18.1)	0.723	Frist trimester 200 ~ 350 Second trimester 200 ~ 400 Third trimester 200 ~ 400
Livestock, poultry, aquatic products and eggs	62 (45.6)	32 (23.5)	42 (30.9)	274 (40.3)	159 (23.4)	247 (36.3)	0.192	Frist trimester 130 ~ 180 Second trimester 150 ~ 200 Third trimester 200 ~ 250
Milk and its products	118 (86.8)	18 (13.2)	0	560 (82.4)	103 (15.1)	17 (2.5)	0.179	Frist trimester 300 Second trimester 300 ~ 500 Third trimester 300 ~ 500
Soybean and nuts	108 (79.4)	1 (0.7)	27 (19.9)	462 (67.9)	10 (1.5)	218 (30.6)	0.004	Frist trimester 25 Second trimester 30 Third trimester 30

Rec: recommended value.

a Below rec: the number and percentage of pregnant women whose food intakes were lower than the recommended value.

b Rec: the number and percentage of pregnant women whose food intakes were within the recommended value.

c Above rec: the number and percentage of pregnant women whose food intakes were higher than the recommended value.

The CDGCI-PW score represented the overall dietary status of pregnant women. The mean CDGCI-PW scores of the policy and control groups were 54.9 and 55.9, respectively, both lower than 60. This indicated poor compliance of pregnant women with the Chinese dietary guidelines for these women. Furthermore, statistical analysis exhibited no significant difference between the two groups (*p* = 0.392).

## 4. Discussion

This study provides snapshots of the anxiety status, physical activity, sleep quality, and dietary status of pregnant women in the rural areas of Guangdong Province and Guangxi Zhuang Autonomous Region from September 2021 to June 2022 (the stage of the dynamic zero COVID-19 policy). As per our understanding, this study is the first to investigate the direct impact of the dynamic zero COVID-19 policy on the anxiety status and lifestyle of pregnant women in rural South China.

The COVID-19 pandemic has undoubtedly resulted in many changes in the life of pregnant women. Paying attention to the impact of prevention and control measures on their life status is also necessary (33). After confounding factors such as maternal age, pre-pregnancy BMI, and chronic history were adjusted, Giesbrecht et al. found that the epidemic increased the incidence of anxiety and depression among Canadian pregnant women (OR = 2.04, *p* < 0.001) (34). In our study of rural South China, 25.7 and 22.4% of pregnant women in the two groups had anxiety disorders, respectively. This proportion was significantly lower than that of fear, anxiety, and depression related to COVID-19 in the Chinese population at the beginning of 2020 (29.6%) (35), which is also lower than that of studies in Canada (56.6%) and Ethiopia (42.1%) (36, 37). The main reason for this phenomenon mainly because infection and mortality rates were significantly reduced towing to the prevention and control measures, such as lockdown and vaccination. You Chuan et al. found that 53.6% of pregnant women in Beijing did not exercise during the epidemic, and only one-fifth of them exercised for 20–60 min every day (38). Unlike pregnant women in urban areas, such as in Beijing, in our study, 29.4 and 22.9% of the pregnant women in the policy and control groups in the studied rural areas had a lower physical activity level, which was close to the physical activity level of rural residents in China (39). A study in Debre Berhan Town, Ethiopia, found that 63% of pregnant women had poor sleep quality during the epidemic, which was mainly a result of sleep rhythm disorder and psychological problems (40). In our study, the proportion of sleep disorders among pregnant women was considerably lower, accounting for only approximately 29% in both the policy and control groups. The study results showed that the dynamic zero COVID-19 policy led to no significant increase in the incidence of anxiety disorders, low physical activity, and poor sleep quality in pregnant women. The implementation of this policy in rural South China was relatively optimistic. A previous study conducted in the urban areas of Greece revealed that pregnant women were most worried about the lack of stability in their living conditions, the economic pressure of the epidemic on their families, and missed social activities because of the imposed restrictions (5). The living conditions of pregnant women in rural South China were relatively stable (41, 42). Under the dynamic zero COVID-19 policy, their economic income and social activities were not particularly affected. In addition, some pregnant women received remote guidance from obstetricians through the network, which also ensured access to maternal health care with the lowest exposure risk (43).

The current impact of the COVID-19 itself and its prevention and control measures on the diet (food type and quantity) of pregnant women is inconsistent. For example, a longitudinal cohort study indicated that the more severe the epidemic, the less the average daily intake of vegetables, fruits, livestock meat, dairy products and nuts by pregnant women (44). However, a few studies have found that the consumption of grains, fruits, vegetables, and dairy products by pregnant women during the epidemic increased significantly compared with that before the epidemic (45). Meanwhile, some researchers have found that changes in the food intake of pregnant women were not associated with the epidemic lockdown (46). In our study, the fruit in-take of the policy group significantly increased, whereas the intake of aquatic products decreased significantly. This was possible because the landform of the rural South China involved in this study was mainly mountainous and hilly, which are rich in fruits. Therefore, these pregnant women could conveniently access fruits. Moreover, the time of our survey did not include summer. The weather was suitable and it was easy to store fruits. However, the local supply of fish, shrimp, shellfish, and other aquatic products was not sufficient. Under the dynamic zero COVID-19 policy, their supply was affected by the logistics blockage. Furthermore, the COVID-19 virus is often detected in aquatic products requiring cold-chain transportation (47). Therefore, pregnant women may also worry about the risk of infection, which thus reduces the intake of aquatic products. Therefore, providing corresponding food supply and organized nutritional support in these rural areas during the future epidemic can be advocated. Different from previous studies (34, 48, 49), our study further evaluated the impact of COVID-19 itself and its prevention and control measures on the dietary quality of pregnant women. The results revealed that the dietary structure of pregnant women in rural South China was unreasonable, and their compliance with the Chinese dietary guidelines for pregnant women was also poor. The epidemic control policy in this study, the dynamic zero COVID-19 policy, had little impact on the overall diet structure of pregnant women. There is an urgent need to provide education on scientific diet and moderate physical activity for pregnant women in these areas of South China to ensure reasonable nutrition intake and weight gain during pregnancy.

This study also had some limitations. First, this study was a cross-sectional survey, so all inferences about causality need to be made cautiously and only associations can be recognized. Second, retrospective questionnaires were used to obtain the data regarding the anxiety status, physical activity, sleep quality, and food intake of pregnant women, which had a certain recall bias. Third, the difficulty of conducting a survey in risk areas had resulted in a relatively small sample size of this study, which may affect the statistical power and precision of the results. Fourth, due to the wide differences in the geographical environment, climate, customs, and medical service levels in different regions of China, our study only investigated pregnant women in the rural areas of Guangdong Province and Guangxi Zhuang Autonomous Region, which do not rep-resent the whole of China. Large sample size studies in additional areas are required to provide more powerful evidence for the impact of COVID-19 on the physical and mental health and lifestyle of pregnant women in China.

## 5. Conclusion

In conclusion, the current epidemic situation in China has greatly improved, but we, especially pregnant women, should not relax our vigilance because they are in a very important physiological stage, and more attention should be paid to the health status and behavior of pregnant women. According to our study, the dynamic zero COVID-19 strategy had little impact on the anxiety status, physical activity, and sleep disorders of pregnant women in the rural areas of South China, but it affected their intake of some food groups. Our results may help provide information for targeted public health strategies to support pregnant women in rural South China during the current epidemic and other similar future public health crises.

## Data availability statement

The raw data supporting the conclusions of this article will be made available by the authors, without undue reservation.

## Ethics statement

The studies involving human participants were reviewed and approved by Ethics Committee of Tongji Medical College, Huazhong University of Science and Technology (No. 2021-S092). The patients/participants provided their written informed consent to participate in this study.

## Author contributions

ZW and YD: conceptualization and design of the work. YD and XS: data analysis and writing—original draft preparation. ZW, YD, and XS: writing—review and editing. XS, GL, QL, ZY, YP, and HD: study implementation and data collection. ZW: supervision and funding acquisition. All authors have read and agreed to the published version of the manuscript.

## Funding

This research was supported by the National Program on Basic Research Project of China (2019FY101001) and a project funded by the Priority Academic Program Development of Jiangsu Higher Education Institutions (Public Health and Preventive Medicine). Neither donor had any role in study design, data collection and analysis, decision to publish, or preparation of the manuscript.

## Conflict of interest

The authors declare that the research was conducted in the absence of any commercial or financial relationships that could be construed as a potential conflict of interest.

## Publisher’s note

All claims expressed in this article are solely those of the authors and do not necessarily represent those of their affiliated organizations, or those of the publisher, the editors and the reviewers. Any product that may be evaluated in this article, or claim that may be made by its manufacturer, is not guaranteed or endorsed by the publisher.
